# Isolated Atypical Pedicle Stress Fractures in Patients on Prolonged Bisphosphonate Therapy: Report of Two Cases and a Literature-Based Proposal for Diagnostic Criteria

**DOI:** 10.3390/jcm14238537

**Published:** 2025-12-01

**Authors:** Maria Auron, Tamar Fisher-Negev, Gal Barkay, Josh E. Schroeder

**Affiliations:** 1 Spine Surgery Unit, Department of Orthopedics, Hadassah Hebrew University Medical Center, Kalman Yaakov Man St., Jerusalem 9112001, Israel; tamar.negev@mail.huji.ac.il (T.F.-N.); galba@hadassah.org.il (G.B.); jes@hadassah.org.il (J.E.S.); 2 School of Pharmacy, Faculty of Medicine, Hebrew University of Jerusalem, Kalman Yaakov Man St., Jerusalem 9112001, Israel

**Keywords:** lumbar vertebrae, atypical fracture, bisphosphonates, osteoporosis, pedicle fracture, stress fracture

## Abstract

**Background/Objectives:** Long-term bisphosphonate (BP) therapy is an effective treatment for osteoporosis but has been associated with rare complications such as atypical femoral fractures (AFFs). Emerging reports suggest that similar insufficiency fractures may also occur in other skeletal sites, including the lumbar pedicles. This study reports two rare cases of isolated bilateral lumbar pedicle stress fractures in patients on prolonged BP therapy. Along with a structured literature review, the objective was to evaluate whether diagnostic criteria derived from those used for AFFs may apply to these atypical vertebral fractures. **Methods:** Two patients with osteoporosis and on long-term BP therapy diagnosed with isolated lumbar pedicle stress fractures were retrospectively analyzed. A structured literature review identified similar reported cases. All cases were evaluated using the 2010 American Society for Bone and Mineral Research AFF criteria to assess applicability to isolated pedicle stress fractures. **Results:** Both patients demonstrated bilateral lumbar pedicle fractures without vertebral body involvement. One was treated conservatively; the other underwent robotic-assisted percutaneous pedicle screw fixation with documented fracture healing at six months. The literature review identified eight similar cases of isolated pedicle fractures in patients on prolonged BP therapy. Clinical course and imaging findings demonstrated stress-type features analogous to AFFs. The proposed AFF-based diagnostic criteria for pedicular insufficiency fractures were found to be applicable to all ten patients. **Conclusions:** Isolated bilateral pedicle stress fractures may represent a spinal analog of AFFs. Based on shared clinical and imaging features, we propose preliminary diagnostic criteria for atypical pedicular insufficiency fractures. Increased awareness and standardized criteria may aid in early diagnosis and reporting, encouraging further studies on this rare spinal entity.

## 1. Introduction

Bisphosphonates (BPs) are widely prescribed for the treatment and prevention of osteoporosis, with well-established efficacy in reducing the risk of osteoporotic fractures [1]. Although generally considered safe, prolonged suppression of bone remodeling by bisphosphonates has been associated with uncommon side-effects, most notably atypical femoral fractures (AFFs) [2,3]. These are diagnosed based on a set of major and minor criteria as described by the Task Force of the American Society for Bone and Mineral Research (ASBMR), including absence of significant trauma, typical localization, delayed healing, and characteristic radiologic features such as transverse orientation and cortical thickening [4,5]. Evidence is emerging that atypical bisphosphonate-associated fractures may also occur in other skeletal regions [6,7]. Lumbar pedicle stress fractures, also referred to as pediculolysis, are uncommon and have been described in the setting of contralateral spondylolysis, prior spinal instrumentation, in association with osteoporotic vertebral compression fractures or as a result of stress-related activities, such as in highly active athletes [8,9,10,11,12,13]. However, isolated pedicle stress fractures without identifiable precipitating factors remain notably rare and poorly understood. These atypical spinal fractures may be an underdiagnosed entity, as patients often present with nonspecific back pain and initial imaging may appear normal. Heightened awareness and improved recognition are crucial to avoid delayed or missed diagnoses.

Recent observations have raised the possibility of a causal relationship between long-term bisphosphonate therapy and atypical pedicle fractures [14,15,16]. The pedicle shares certain biomechanical characteristics with long bones, including a load-bearing function and a predominantly cortical structure, which may render it susceptible to insufficiency or stress fractures. The criteria used to define atypical femoral fractures may also be applicable to atraumatic isolated pedicle fractures with similar clinical and radiographic features, suggesting a potentially shared underlying pathophysiology [17].

We present two cases of isolated lumbar pedicle stress fractures in osteoporotic patients treated with bisphosphonates for over five years. These cases demonstrate clinical and imaging features resembling atypical femoral fractures (AFFs) and suggest a possible spinal manifestation of bisphosphonate-related effects. We hypothesized that the diagnostic criteria for AFFs could be adapted to define these atypical pedicular fractures. We review the literature to evaluate whether AFF-based diagnostic features can be applied to atypical pedicle fractures. The aim of this study is to raise awareness of this uncommon entity and to encourage consideration of AFF-derived criteria as a framework for diagnosis.

## 2. Materials and Methods

This study is a retrospective analysis of two patients diagnosed with isolated lumbar pedicle stress fractures at our institution. Clinical and imaging data were obtained from hospital electronic medical records. Cases were reviewed for demographics, comorbidities, bisphosphonate treatment, clinical presentation and details of diagnostics and treatment. Available imaging, including plain radiographs, computed tomography (CT), and magnetic resonance imaging (MRI), was analyzed for fracture location and morphology. Fracture union was assessed by follow-up CT when available. All imaging was interpreted by two fellowship-trained spine surgeons in a non-blinded manner. This case report was prepared according to the CARE guidelines where applicable [18]. Approval from the institutional ethics committee was waived due to the retrospective nature of the study and the use of anonymized data. A structured literature search was conducted using the PubMed, Scopus and Google Scholar databases using combinations of the following keywords: “bisphosphonate”, “alendronate”, “risedronate”, “zoledronate”, “zoledronic acid”, “pedicle”, “posterior element”, “spine”, “stress fracture”, “insufficiency fracture”, and “atypical fracture”. No time or language restrictions were applied. Only original clinical studies involving isolated pedicle fractures in human subjects with long-term bisphosphonate exposure were included. Reference lists of the studies included were subsequently screened for additional relevant publications.

## 3. Results

### 3.1. Case Presentation 1

An 81-year-old female presented with severe left-sided radicular leg pain and markedly reduced walking tolerance. Her medical history was notable for osteoporosis, with no prior spinal fractures, trauma, or spinal surgery. Dual-energy X-ray absorptiometry (DEXA), performed 10 months prior to presentation, showed a femoral neck T-score of −2.3 and a lumbar spine T-score of −1.1. She had completed a 5-year course of intravenous zoledronate (5 mg annually), which had been discontinued one year prior. She was started on daily subcutaneous teriparatide (20 µg) three months before presentation to our clinic. Her body mass index (BMI) was 21.

**Figure 1:**
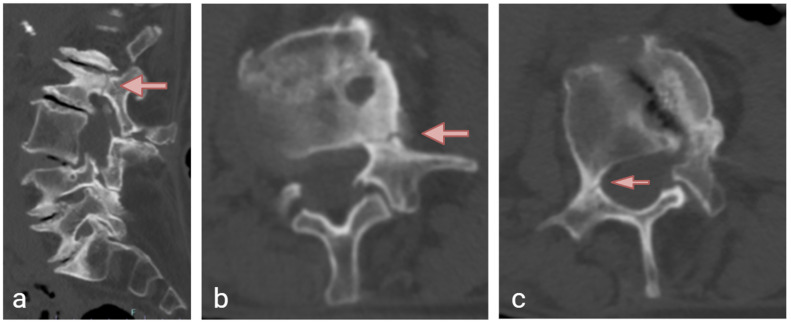
Computed tomography (CT) scan showing a left pedicle fracture of the L2 vertebra in the (**a**) sagittal and (**b**) axial view. The fracture line (arrow) is characterized as transverse to the longitudinal axis of the pedicle. There is an incomplete short oblique fracture (arrow) of the right L2 pedicle on axial view (**c**). Bilateral cortical thickening (arrow) of the pedicles and periosteal reaction of the left pedicle can be observed.

**Figure 2:**
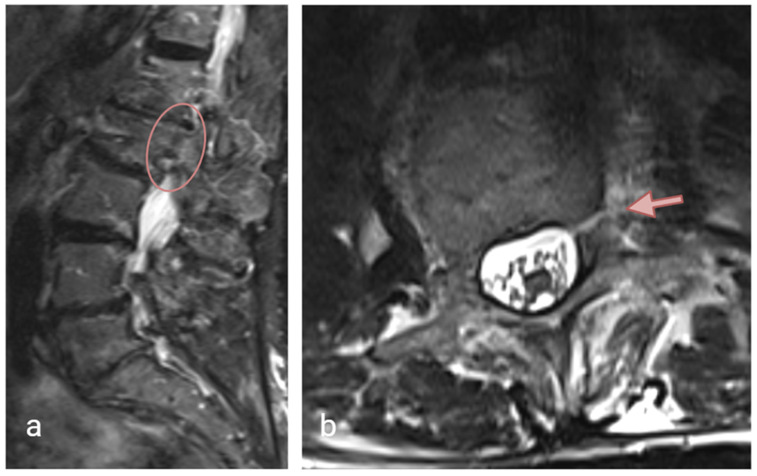
MRI at 3 months; (**a**) sagittal T2/STIR sequence and (**b**) axial T2W images demonstrate a visible fracture line at the left L2 pedicle.

Physical examination revealed tenderness over the lumbosacral region, without motor weakness or sensory deficits in the lower extremities. A non-contrast computed tomography (CT) scan of the lumbar spine demonstrated mild degenerative lumbar scoliosis, a left pedicle fracture and an incomplete right pedicle fracture of the L2 vertebra (Figure 1). No vertebral body fracture was found throughout the lumbar spine. MRI excluded malignant or infectious etiologies. The mean Hounsfield unit (HU) density, using the L3 trabecular region of interest (ROI) measurement [19], was 123. A non-operative approach was initially selected, and MRI at 3 months revealed a hypointense fracture line within the hyperintense marrow edema on T2-STIR (short tau inversion recovery) weighted images, indicating incomplete healing (Figure 2). The patient was unfortunately lost to follow-up after the initial visit; treatment options could therefore not be discussed, and no further imaging was available.

### 3.2. Case Presentation 2

**Figure 3:**
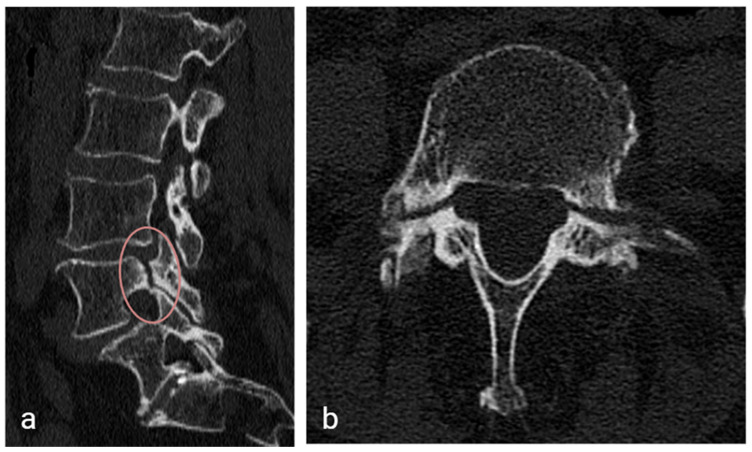
CT images showing bilateral L4 pedicle stress fractures with short oblique fracture orientation at L4–L5 and pedicle sclerosis. (**a**) Sagittal view. (**b**) Axial view.

A 75-year-old woman presented with acute low back pain radiating to the left calf, which began four days earlier after sneezing. She had no previous spinal fractures, trauma or surgery. She had a history of osteoporosis with prior bilateral proximal humerus fractures, and had been treated with oral bisphosphonates for five years (four years with alendronate, one year with risedronate). Her most recent DEXA showed T-scores of −1.0 (femoral neck) and −2.8 (spine) one year before presentation. On examination, she walked with an antalgic limp and had tenderness over the lumbosacral spine. Her neurological status was normal. CT revealed bilateral L4 pedicle stress fractures with grade 1 spondylolisthesis at L4–L5, without vertebral body involvement (Figure 3). No vertebral body fractures were seen in the lumbar spine, and the L3 mean HU value was 85. Laboratory parameters including Vitamin D (25-hydroxycholecalciferol = 42 ng/mL), C-reactive protein (CRP = 0.83 mg/dL), serum calcium (Ca = 8.7 mg/dL), serum phosphorus (P = 4.2 mg/dL), intact parathyroid hormone (PTH = 8.4 pmol/L), thyroid stimulating hormone (TSH 0.62 mU/L) and serum magnesium (Mg = 1.9 mg/dL) were within normal limits.

**Figure 4:**
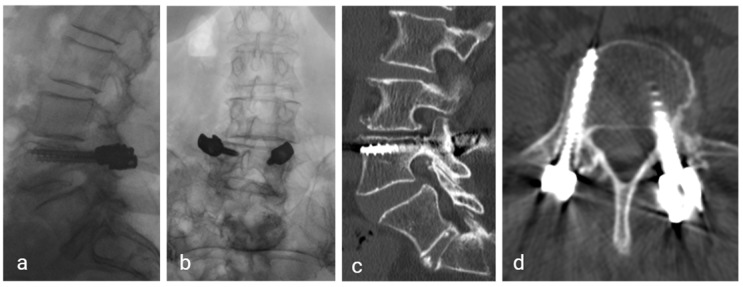
Postoperative follow-up at 6 months, demonstrating stable grade 1 spondylolisthesis and fracture consolidation. (**a**) Sagittal and (**b**) axial radiograph. (**c**) Sagittal and (**d**) axial CT showing the bicortical trajectory of the pedicle screws.

After discussing the options of conservative versus surgical treatment, the patient consented to surgical fixation. She underwent robotic-assisted (Mazor X, Medtronic, Minneapolis, MN, USA) percutaneous fixation with bilateral bicortical L4 pedicle screws to achieve compression of the fractures. Postoperative recovery was uneventful, and she was discharged home on postoperative day 3. Risedronate was discontinued and substituted with teriparatide. Follow-up over six months showed progressive pain resolution and fracture consolidation on CT (Figure 4).

### 3.3. Structured Literature Review

A review of the existing literature was performed to identify similar cases and evaluate potential links between bisphosphonate use and atypical pedicle fractures. The electronic literature search conducted in November 2025 yielded a total of 13 records. After removal of 2 duplicates and identification of 2 additional records, 13 articles were screened by title and abstract. Eight articles met the inclusion criteria and underwent full-text review. All included articles were single case reports. A summary of the 8 cases included in the literature review, as well as the two cases of the present study, is presented in Table 1. Of all 10 cases, including the 2 from our institution, 9 patients (90%) were female, with a mean age of 70.8 years (SD ± 7.9, range: 61–82). A total of 13 fractured vertebrae were identified, with L5 being the most frequently affected level (*n* = 4, 30.8%), followed by L3 and L4 (*n* = 3, 23.1% each), L2 (*n* = 2, 15.4%), and L1 (*n* = 1, 7.7%). The mean duration of bisphosphonate therapy was 6.0 years (range 3–10 years, SD ± 2.66). Alendronate was the most commonly used bisphosphonate (50%), followed by risedronate (30%), with ibandronate, minodronate, and zoledronate each used in 10% of patients. Six patients were managed conservatively, and four underwent surgical fixation. Fracture union was documented in 7 cases (70%), verified by postoperative CT in 6 cases and X-ray in 1 case.

### 3.4. Diagnostic Criteria

The American Society of Bone and Mineral Research Task Force 2010 defined a set of major and minor criteria for defining atypical femur fractures (AFFs). By their definition, all major features, including (1) location along the femoral diaphysis, (2) transverse or short oblique orientation, (3) minimal or no trauma preceding the fracture, (4) a medial spike when the fracture is complete and (5) absence of comminution, were required to be present to define a femoral fracture as atypical. None of the minor features were required for diagnosis but have been associated with these fractures. These included localized cortical thickening, periosteal reaction of the lateral cortex, prodromal pain, bilateral involvement, delayed healing, prolonged BP exposure (usually ≥3–5 years) or other antiresorptives, glucocorticoids, or proton pump inhibitors [2,23].

Based on our hypothesis that the diagnostic criteria for AFFs could be adapted to identify atypical pedicular fractures potentially linked to prolonged bisphosphonate use, we derived a preliminary set of criteria modeled on the AFF definition and evaluated their applicability to all reviewed cases (Table 2). We found that all of the proposed Major diagnostic criteria were applicable to our two reported patients as well as most of the eight patients previously published in the literature.

We suggest that all five Major Features must be present to designate a pedicle fracture as atypical and to distinguish it from the more common osteoporotic vertebral compression fractures. Major criteria include (1) isolated vertebral pedicle fracture; (2) absence of trauma or minimal trauma; (3) a transverse or short oblique fracture orientation; (4) non- or minimally comminuted fracture and (5) complete bilateral pedicle fracture, or complete unilateral and incomplete contralateral fracture. Minor criteria are not required but support the diagnosis when present: localized periosteal reaction, sclerotic or corticalized pedicle, prodromal symptoms of low back pain, multiple-level pedicle fracture or concomitant atypical fracture at another site, or signs of delayed or incomplete healing. Long-term bisphosphonate treatment should be recognized in this association. Specifically excluded are fractures of the pars interarticularis, fractures of the vertebral body extending into the pedicle, pathological fractures associated with primary or metastatic bone tumors, and peri-implant fractures.

## 4. Discussion

This study highlights two rare cases of isolated bilateral pedicle stress fractures in elderly patients receiving long-term BP treatment. These cases, with previously published similar cases, suggest that atypical pedicular insufficiency fractures may represent a rare manifestation potentially associated with prolonged bisphosphonate therapy, similar to AFFs. We propose a set of AFF-derived diagnostic criteria intended to aid in the recognition and standardized reporting of atypical pedicle fractures. Early identification of this rare pathology is crucial to allow timely intervention and help prevent potential disease progression, pain and disability.

While osteoporotic vertebral body compression fractures present a significant health problem worldwide [24], the pedicles are a highly uncommon site of osteoporosis-related fractures. The neural arch primarily resists and transmits posterior element loads, including compressive, shear, bending, and torsional forces during spinal motion. Mechanical testing of the neural arch has identified the pedicle as the second weakest part after the pars interarticularis [25]. The pedicle has been described as a strong bridge between the posterior elements and vertebral body, composed primarily of cortical bone surrounding a cancellous core, analogous to a long bone [26]. Stress or insufficiency type fractures occur when mechanical loading exceeds the bone’s biological capacity to heal, leading to microstructural fatigue and failure [27]. Pedicle stress fractures are extremely rare, and have most frequently been reported in association with osteoporotic vertebral compression fractures, contralateral spondylolysis, spondylolisthesis or prior spinal surgery [8,10,11,28,29,30,31,32,33,34], where altered load transmission might precipitate pedicle failure. Additionally, cases of pediculolysis have been reported in young individuals involved in repetitive strenuous activity [13,35,36,37,38]. Isolated pedicle stress fractures without clear identifiable etiology are anecdotal and poorly understood [39,40,41].

Current evidence suggests that atypical femoral fractures represent stress- or insufficiency-type fractures, and they have been linked to long-term bisphosphonate treatment [2,5,23]. In 2010, the American Society for Bone and Mineral Research (ASBMR) developed a case definition for AFFs, based on major and minor features including typical location, fracture morphology and absence of significant trauma [2]. The report was later revised in 2014 to emphasize key radiographic features that distinguish these fractures from the more common osteoporotic femoral diaphyseal fractures [5]. Morphologically, AFFs appear to originate as stress fractures on the lateral cortex of the femur, a region subjected to high tensile bending forces [42], similar to the forces acting on the vertebral pedicles during spinal flexion and extension.

While bisphosphonates are beneficial for preventing osteoporotic fractures, they may impair the healing of stress fractures, allowing progression from incomplete to complete fractures. They act by suppressing bone turnover by inhibiting osteoclastic activity, leading to increased mineralization and strength, but reduced microdamage repair. These changes reduce the energy-absorbing capacity of bone and may predispose to insufficiency fractures [2,43,44]. In AFFs, the transverse fracture morphology, lack of comminution and typically thickened cortices suggest tensile forces on hard but brittle bone [2,5]. Attempts have been made to investigate the potential role of bisphosphonates in patients with AFFs by measuring bone turnover markers (e.g., serum or urine N- or C-telopeptide) [45]. However, the results have often been non-informative, with markers falling within the reference range in up to 79% of cases [23,44]. An epidemiological association has, however, been found between AFFs and treatment with BPs for prolonged times, usually 3 to 5 years [2,23,46]. This suggests a role of BPs in the pathogenesis of atypical femoral fractures, although such fractures have also been reported in individuals with no exposure to the drug [2].

Several case reports have described bilateral pedicle fractures in patients with prolonged bisphosphonate exposure. El Rachkidi et al. were the first to report an isolated bilateral pedicle fracture linked to long-term bisphosphonate therapy. Their patient, a 66-year-old woman on risedronate for 10 years, sustained atraumatic bilateral L5 pedicle fractures with no other evident etiology [16]. Karabay et al. described multi-level bilateral pedicle fractures in a woman with mild osteoporosis and bisphosphonate use [20], while Kim et al. reported bilateral L4 pedicle fractures occurring alongside bilateral AFFs after three years of intravenous BP therapy [7]. Subsequent reports further reinforced the association [14,21]. In their report, Theodorakis et al. emphasized that these rare fractures closely mirror the features of AFFs, and they first advocated for these diagnostic criteria to classify bisphosphonate-associated pedicle fractures [17]. Most recently, Fujita et al. reported a case of bilateral pedicle fracture in an elderly patient after 9 years of alendronate treatment [22].

These previously published cases suggest that an analogous pathophysiological process to AFFs may occur in the vertebral pedicles, resulting in atypical stress fractures. While the pedicle is not a weight-bearing long bone, its cortical architecture and exposure to repetitive tensile and shear forces during spinal motion may render it vulnerable to stress-related failure, possibly influenced by the suppressed bone turnover of prolonged BP exposure. Given the absence of significant trauma or other evident etiology, together with shared radiological features, such as bilaterality, transverse or short oblique orientation, and minimal comminution, most of the fractures in our analysis showed features consistent with the ASMBR major criteria for AFFs. In addition, we noted findings corresponding to minor AFF criteria, including sclerotic or corticalized pedicles, local periosteal reaction, and delayed healing, in several cases. These parallels support the rationale for cautiously extending AFF-derived diagnostic criteria to atypical pedicular insufficiency fractures, particularly in the light of the prolonged BP exposure of the reported cases (mean duration of 6.0 years).

Biomechanical factors may modulate the risk of pedicle stress fractures. Biomechanical studies supported by clinical reports have shown that unilateral spondylolysis increases stress on the contralateral pedicle [35,47,48], possibly predisposing it to stress fractures. Similarly, prior spinal fusion or decompression can disrupt normal biomechanics through segmental instability and force redistribution, with pedicle fractures reported at adjacent or instrumented levels [28,49,50,51]. Fractured pedicles have also been observed adjacent to osteoporotic vertebral compression fractures [12,30], suggesting that changes in spinal alignment and loading may act synergistically with impaired bone quality. In degenerative lumbar scoliosis, pedicle morphological asymmetry has been noted, with increased cortical thickness and a higher cortical ratio on the concave side [52]. In our two cases, one patient had mild degenerative scoliosis and the other had grade 1 spondylolisthesis, both potentially contributing to asymmetric loading and localized pedicle stress. These mechanical factors may have acted alongside suppressed bone turnover from prolonged BP use, both of which could have contributed to fracture risk. However, the relative contribution of mechanical versus metabolic factors in the pathogenesis of these rare fractures remains unclear, and further research is needed.

From a diagnostic standpoint, recognizing an atypical pedicle fracture can be challenging. Patients typically present with nonspecific back pain, and conventional X-rays are often unremarkable. CT is useful for diagnosing pedicle fractures and differentiating them from arthritis, infection, or neoplasia [40]. As in AFFs, prodromal pain may correspond to early stress reactions detectable by MRI, bone scan or SPECT-CT [53]. MRI may reveal periosteal and endosteal edema, which can indicate an impending stress fracture [4]. We recommend that advanced imaging should be considered in osteoporotic patients on long-term bisphosphonates with back- or radicular pain, and a high level of diagnostic vigilance is required to avoid missed or delayed diagnosis.

Given that published cases are few, no standardized treatment guideline exists for atypical pedicle fractures. In our second case, the patient underwent surgery guided by our experience and the principles of AFF management, which recommend surgical stabilization of complete and incomplete fractures [4]. Reported approaches for atypical pedicle fractures have ranged from conservative management with or without bracing [16,21], to surgical intervention ranging from minimally invasive single-level pedicle screw fixation to multi-level lumbar fusion [41,54]. Bisphosphonate therapy should be re-evaluated in this setting, typically by discontinuing the drug and considering alternative osteoporosis treatments such as teriparatide [4,44,55]. Minimally invasive percutaneous pedicle screw fixation offers stabilization with less soft tissue damage and faster recovery than open surgery. Theodorakis et al. employed percutaneous short-segment pedicle screw fixation, allowing early mobilization and symptom relief [17]. Based on the limited available evidence, surgical fixation may be preferred in symptomatic atypical pedicle fractures, particularly when modeled on treatment principles for AFFs [4]. We used robotic assistance to improve screw placement accuracy, optimize trajectory planning for a bicortical lag screw effect, and reduce radiation exposure. This enabled targeted stabilization of the fractures while preserving adjacent segment mobility. CT showed early bony fusion at 6 months, suggesting a favorable effect of this minimally invasive technique. Future studies should aim to establish a treatment algorithm of atypical pedicle fractures and assess the potential benefits of minimally invasive fixation techniques in their management.

This report has several limitations due to the retrospective nature of case analysis and small number of published cases. The small sample size introduces potential selection bias and limits statistical power and generalizability of our findings. Initial plain radiographs and comprehensive imaging were unavailable in both cases, and follow-up of Case 1 was incomplete. Bone biopsies and bone turnover markers were also not available. These factors limit our ability to understand the natural course and pathogenesis of atypical pedicle fractures, including the potential role of bisphosphonates and biomechanical contributors such as spinal alignment. Future multicenter studies are needed to enable detailed histopathology- and imaging-based analyses to increase the understanding of these extremely rare fractures, and to assess the validity of the proposed diagnostic criteria with the aim of generating evidence-based recommendations for prevention and treatment.

## 5. Conclusions

To conclude, isolated bilateral pedicle stress fracture is a very rare entity. We report two such cases in osteoporotic patients on long-term bisphosphonate treatment and propose diagnostic criteria modeled after AFF definitions. While a causal link with prolonged BP exposure remains unproven, increased clinical vigilance and systematic reporting are essential. Further studies are needed to establish prevalence, risk factors, and management strategies for these rare spinal fractures.

## Figures and Tables

**Table 1:** Reported cases of bilateral pedicle insufficiency fractures in patients on long-term bisphosphonate therapy [7,14,15,16,17,20,21,22].

Author (Year)	Age	Sex	Comorbidities	BP Treatment	Fracture Level	Symptom Duration	Treatment	Fracture Union
El Rachkidi (2011) [16]	66	Female	none	10 y risedronate	L5	3 mo	Conservative	Yes (CT 4 mo)
Karabay (2015) [20]	61	Female	OP	4 y alendronate	L1–L4	NR	Conservative	NR
Kim (2015) [7]	64	Female	OP, psoriatic arthritis	3 y ibandronate	L4	NR	Conservative	NR
Surur (2018) [21]	79	Female	OP, s/p chondrosarcoma, colon cancer	10 y risedronate	L5	6 weeks	Conservative	Yes (CT 2 y)
Meleger (2020) [15]	63	Male	OP	3 y 5 mo alendronate	L5	NR	Conservative	Yes (CT 6 mo)
Karasawa (2023) [14]	66	Female	OP, DM	6 y minodronate	L5	2 mo	Surgical: TLIF L5-S1	Yes (CT 2 y)
Theodorakis (2024) [17]	71	Female	OP	5 y alendronate	L3	4 mo	Surgical: L3–4 pedicle screw fixation	Yes (CT 6 mo)
Fujita (2024) [22]	82	Female	OP	9 y alendronate	L3	1 mo	Surgical: L1–L4 pedicle screw fixation	Yes (3 mo)
Case 1	81	Female	OP	5 y zoledronate	L2	NR	Conservative	NR
Case 2	75	Female	OP	4 y alendronate, 1 y risedronate	L4	4 days	Surgical: L4 pedicle screw fixation	Yes (CT 6 mo)

Abbreviations: BP = bisphosphonate; y = years, mo = months; CT = computed tomography; OP = osteoporosis; NR = not reported; DM = diabetes; TLIF = transforaminal lumbar interbody fusion.

**Table 2:** Proposed diagnostic criteria for atypical pedicular fractures (APFs), adapted from the 2010 ASBMR Task Force Case Definition of AFFs [2], and comparison with all reported cases [7,14,15,16,17,20,21,22].

Major Criteria for AFF	Atypical Pedicular Fracture	El Rachkidi (2011) [16]	Karabay (2015) [20]	Kim (2015) [7]	Surur (2018) [21]	Meleger (2020) [15]	Karasawa (2023) [14]	Theodorakis (2024) [17]	Fujita (2024) [22]	Case 1	Case 2
Absence of Trauma or minimal Trauma	Absence of Trauma or minimal Trauma	x	x	x	x	x	x	x	x	x	x
Femoral fracture in any diaphyseal part	Isolated pedicle fracture	x	x	x	x	x	x	x	x	x	x
Transverse or short oblique fracture	Transverse or short oblique fracture	x	x	x	x	x	x	x	x	x	x
Non-comminuted fracture	Non-comminuted fracture	-	x	x	x	x	x	x	x	x	x
Complete fractures extend through both cortices, incomplete only the lateral cortex	Complete bilateral fracture, or complete uni- + incomplete contralateral fracture	x	x	x	x	x	x	x	x	x	x
**Minor criteria**	**Minor criteria**										
Localized periosteal reaction	Localized periosteal reaction	-	x	NR	NR	NR	x	x	NR	x	x
Increase in the thickness of the cortex	Sclerotic/corticalized pedicle	x sclerosis	x	NR	NR	NR	x sclerosis	x corticalized	NR	x sclerosis	x
Prodromal symptoms	Prodromal symptoms	3 mo	NR	x	6 weeks	NR	2 mo	4 mo	1 mo	NR	-
Bilateral fractures	Multiple level pedicle fracture or concomitant AF at other site	-	multilevel	Multisite (AFF + pedicle fracture)	-	-	-	-	-	-	-
Delayed healing	Delayed healing	CT consolidation 4 mo	NR	NR	CT 2 y healing	x CT 6mo pseudoarthrosis	CT 2 y healing	x delayed union, surgery at 4 mo	- (surgery)	NR	- (surgery)

Abbreviations: ASBMR = American Society for Bone and Mineral Research; AFF = atypical femur fracture; AF = atypical fracture; NR = not reported; mo = months; CT = computed tomography.

## Data Availability

The data presented in this study are available on reasonable request from the corresponding author due to local data protection laws and protecting confidentiality.

## Abbreviations

The following abbreviations are used in this manuscript:

AFF	Atypical femoral fracture
BP	Bisphosphonate
ASBMR	American Society for Bone and Mineral Research
CT	Computed tomography
MRI	Magnetic resonance imaging
DEXA	Dual-energy X-ray absorptiometry
BMI	Body mass index
HU	Hounsfield unit
ROI	Region of interest
STIR	Short tau inversion recovery
OP	Osteoporosis
DM	Diabetes
TLIF	Transforaminal lumbar interbody fusion
AF	Atypical fracture
SPECT	Single-photon emission computed tomography
